# Clinical Forms of Japanese Spotted Fever from Case-Series Study, Zigui County, Hubei Province, China, 2021

**DOI:** 10.3201/eid2901.220639

**Published:** 2023-01

**Authors:** Zhongqiu Teng, Ping Gong, Wen Wang, Na Zhao, Xiaojing Jin, Xiangrong Sun, Haijian Zhou, Junlin Lu, Xuebing Lin, Bohai Wen, Biao Kan, Jianguo Xu, Tian Qin

**Affiliations:** State Key Laboratory of Infectious Disease Prevention and Control, Beijing, China (Z. Teng, W. Wang, N. Zhao, X. Jin, H. Zhou, B. Kan, J. Xu, T. Qin);; National Institute for Communicable Disease Control and Prevention, Beijing (Z. Teng, W. Wang, N. Zhao, X. Jin, H. Zhou, B. Kan, J. Xu, T. Qin);; Zigui People's Hospital, Yichang, China (P. Gong, J. Lu, X. Lin);; Nanchang Municipal Center for Disease Control and Prevention, Nanchang, China (X. Sun);; Beijing Institute of Microbiology and Epidemiology, Beijing (B. Wen)

**Keywords:** Rickettsia japonica, Japanese spotted fever, vector-borne infections, tickborne illnesses, rickettsia, febrile illnesses, fatal outcomes, cytokines, China, bacteria

## Abstract

We report a case-series study of 5 patients with Japanese spotted fever from the Three Gorges Area in China, including 1 fatal case. Seroprevalence of *Rickettsia japonica* was ≈21% among the local population. Our report highlights the emerging potential threat to human health of Japanese spotted fever in the area.

Japanese spotted fever (JSF), caused by the bacterium *Rickettsia japonica*, was first described in 1984 in Japan (*1*). It has been recognized in multiple countries in Asia, including Japan, South Korea, the Philippines, Thailand, and China (*1*–*5*), suggesting that it is endemic in Asia. In China, *R. japonica* has been detected in ticks from the central, southeast, and northeast regions (*6*–*8*). Since JSF cases were first found in Anhui Province in 2013 (*5*), a total of 39 have been reported in the Dabie Mountains on the borders of Henan, Anhui, and Hubei Provinces and in the Tianmu Mountains in Zhejiang Province on the eastern coast (Appendix Figure 1) (*9*–*11*).

## The Study

Our study was approved by the ethics committees of the National Institute for Communicable Disease Control and Prevention (ICDC). During April–October 2021, five JSF cases were found in Zigui County, Hubei Province, in the Three Gorges area of China (Appendix Figure 1), including case 1, in which the patient died because of multiorgan failure. Among the 5 case-patients (3 female, 2 male), disease onset occurred during April–October (2 cases in spring, 3 in autumn); median age of case-patients was 53 years (range 47–70 years) (Table 1). All had histories of activity in fields, but none was aware of any tick bites. All initially manifested signs and symptoms of sudden fever (≥38.5°C), headache, chills, fatigue, chest tightness, shortness of breath, and dyspnea. All had rash and eschar (Appendix Figure 2), except for case-patient 1, who had no eschar. Case-patients 1, 3, and 5 experienced vomiting and case-patient 3 had abdominal pain. Medical laboratory tests (Table 2) showed that case-patients 1, 3, and 5 had thrombocytopenia and lymphopenia, case-patients 1 and 5 had neutrophilia, and case-patients 1 and 4 had mild anemia. All 5 patients had remarkably elevated levels of C-reactive protein and D-dimer, and except for case-patient 5, all had elevated liver-associated enzymes. Proteinuria was detected in case-patients 1, 2, 3, and 5. Case-patients 1, 3, and 5 had simultaneous edema and effusion and case-patient 5 showed multiple effusions (pelvic, pleural, pericardial, and ascites). 

**Table 1:** Epidemiologic and clinical characteristics of patients with *Rickettsia japonica* infection, Zigui County, Hubei Province, China

Characteristics	Case 1*	Case 2	Case 3	Case 4	Case 5
Sex	Female	Male	Male	Female	Female
Age, y	58	47	68	57	70
Month of admission	Apr	Jun	Sep	Sep	Oct
Occupation	Farmer	Village teacher	Farmer	Farmer	Farmer
Possible causative exposures	Picking tea, tea garden	Collecting bamboo, bamboo groves	Herding sheep, woodland	Farm work, fields	Farm work and chopping wood, fields and woodland
Previous illness	Healthy	Healthy	Hypertension	Healthy	Hypertension
Day of admission†	8	4	6	15	5
Signs and symptoms					
Highest temperature, °C	39.0	39.0	39.0	38.5	39.5
Fever type	Continued	Continued	Continued	Undetermined‡	Undetermined§
Headache	Present	Present	Present	Present	Present
Malaise	Present	Present	Present	Present	Present
Myalgia	Present	Present	Present	Present	Present
Chills	Present	Present	Present	Present	Present
Eschar	Absent	Present	Present	Present	Present
Rash (day)	Present (11)	Present (3)	Present (10)	Present (13)	Present (10)
Hypotension (day)	Present (9)	Absent	Absent	Absent	Absent
Dyspnea (day)	Present (9)	Present (6)	Present (10)	Present (15)	Present (5)
Vomiting (day)	Present (9)	Absent	Present (10)	Absent	Present (7)
Edema (day)	Face (9)	Absent	Lower extremities (10)	Absent	Lower extremities (10)
Clouding of consciousness	Present	Present	Present	Present	Present
Proteinuria	+++	+–	+	–	**++**
Anuria (day)	Present (9)	Absent	Absent	Absent	Absent
Abdominal pain	Absent	Absent	Present (11)	Absent	Absent
Pelvic effusion	Absent	Absent	Absent	Absent	Present (5)
Pleural effusion (day)	Present (9)	Absent	Present (10)	Absent	Present (5)
Pericardial effusion	Absent	Absent	Present (10)	Absent	Present (5)
Ascites effusion	Absent	Absent	Absent	Absent	Present (5)

*Fatal case. †Day on which sign or symptom manifested in patient, when applicable. The day when the patients first experienced headache and fatigue was defined as Day 0. ‡Difficulty determining fever type because of drug intervention. §Urine protein concentration: –, <0.1 g/L; +–, 0.1–0.2 g/L; +, 0.2–1.0 g/L; ++, 1.0–2.0 g/L; +++, 2.0–4.0 g/L.

**Table 2:** Laboratory findings of the patients with *Rickettsia japonica* infection, Zigui County, Hubei Province, China*

Laboratory findings*	Reference value	Case 1	Case 2	Case 3	Case 4	Case 5
Leukocyte, × 10^9^ cells/L	4.0–10.0	9.09	3.50	7.78	8.45	9.15
Hemoglobin, g/L	110–150 (F), 120–160 (M)	109	130	127	105	115
Platelets, × 10^9^/L	100–300	45	99	72	110	82
Lymphocytes, × 10^9^ cells/L	0.8–3.5	0.52	0.72	0.91	2.29	0.64
Neutrophils, × 10^9^ cells/L	1.8–6.3	8.11	2.55	5.80	5.80	7.90
CRP, mg/L	0.8–8.0	199.53	72.8	77.00	72.00	111.72
PCT, μg/L	<0.5	5.64	0.38	2.14	0.4	0.54
D-dimer, μg/mL	0–0.5	4.200	2.081	2.160	1.090	1.080
TBil, mg/L	3–13	38.0	12	12	9.3	11
DBil, mg/l	0–2.5	24.0	2.0	3.4	2.7	2.0
TBA, μmol/L	<10	43.1	3.9	8.6	4.8	4.9
TP, g/L	60–80	48.7	62.9	52.3	58.2	65
Albumin, g/L	35–55	23.8	36.2	30.6	35.6	35.9
A/G	1.5–2.5	1.0	1.4	1.4	1.6	1.2
ALT, U/L	0–35	21	69	177	167	16
AST, U/L	0–40	114	88	167	111	29
BUN, mmol/L	2.9–7.5	7.81	3.81	10.80	2.38	7.54
Creatinine, mg/L	40–100 (F), 50–110 (M),	93.2	84.9	74.1	58.2	83.9
Uric acid, μmol/L	150–357 (F), 200–416 (M)	378	301	259	182	210
LDH, U/L	100–300	587	412	403	433	368
Creatine kinase, U/L	18–198	257	186	51	48	125
α-HBDH, U/L	90–220	401	294	254	317	323
Sodium, mmol/L	135–145	137.7	141.9	132.9	131.4	136.5

*α-HBDH, α-hydroxybutyrate dehydrogenase; A/G, albumin/globulin ratio; ALT, alanine aminotransferase; AST, aspartate aminotransferase; BUN, blood urea nitrogen; CRP, C-reactive protein; DBil, direct bilirubin; LDH, lactate dehydrogenase; TBA, total bile acids; TBil, total bilirubin; TP, total protein.

Most laboratory tests were flagged as abnormal for case-patient 1, who died. She received an initial antimicrobial treatment (Appendix) at the village clinic without a causative agent being identified and already had severe sepsis (sequential organ failure assessment score = 12) by the time she was admitted to the hospital on day 8 after symptom onset. Disseminated intravascular coagulation was diagnosed, and she died of multiple organ failure on day 9 despite continuous intensive therapy. The other 4 patients recovered without sequelae after receiving doxycycline or minocycline treatment (Appendix). 

Using a PCR assay, we detected 6 rickettsial genes (*groEL*, *glt*A, *omp*A, *omp*B, *sca*4, and 17kDa gene) in a blood specimen from case-patient 1. In addition, we amplified *groEL*, *glt*A, and 17kDa genes from case-patients 2, 3, and 5 but only *glt*A and 17kDa from case-patient 4. For all 5 patients, the genomic sequences of each amplified target gene were 100% identical to each other; phylogenetic analysis revealed that the causative agent was most closely related to *R. japonica* (Figure). We submitted the obtained sequences to GenBank (accession nos. OM966424–8 for *glt*A, OM966422 for *omp*A, OM966423 for *omp*B, OM966412–6 for 17kDa, OM966417 for *sca*4, and OM966418–21 for *groEL*). We obtained 1 stable rickettsial isolate, designated *R. japonica* strain YC21, from the blood of case-patient 1 using Vero cell cultures (Appendix Figure 3) and obtained the whole genomic sequence from the isolate (GenBank BioProject PRJNA812951). Phylogenetic analysis based on core genes suggested that *R. japonica* strain YC21 was most closely related to *R. japonica* strain LA16/2015 (Appendix Figure 4); 30 virulence-associated genes of *R. japonica* strain YC21 (Appendix Table 2), predicted using the virulence factor database (http://www.mgc.ac.cn/VFs), were completely homologous to those of strain LA16/2015.

**Figure:**
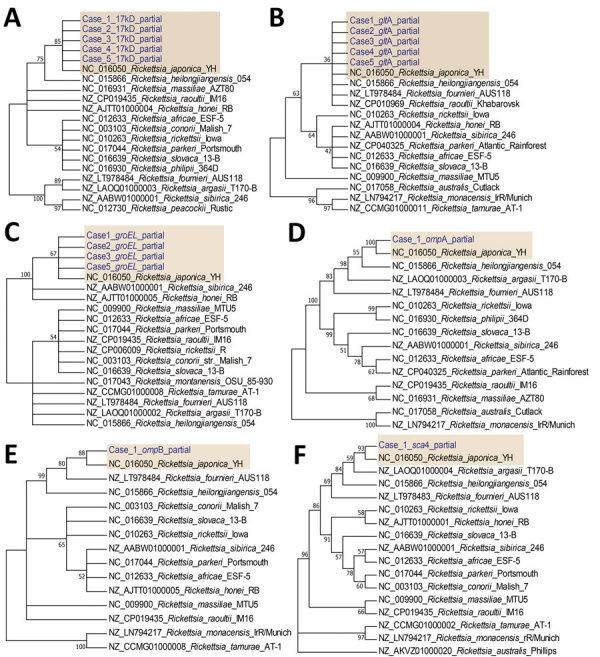
Bootstrap consensus phylogenetic tree constructed based on partial sequences of 17kDa (A), *glt*A (B), *groEL* (C), *omp*A (D), *omp*B (E), and *sca*4 (F) amplified from blood specimens from 5 spotted fever patients in Japan (yellow shading). We aligned sequences using MUSCLE within MEGA6 software (http://www.megasoftware.net). We analyzed phylogenetic relationships using the neighbor-joining method with 1,000 bootstrap replicates; boot values are shown next to the branches. Genbank accession numbers for the *Rickettsia* strains retrieved are indicated.

We used an immunofluorescence assay using *R. japonica* strain YC21 as coating antigen (a cutoff of 1:64 was determined by testing negative and positive samples) and *R. rickettsii* (Focus Diagnostics, http://focusdiagnostics.in) to test serum-specific antibodies from the 5 JSF patients and 100 healthy subjects recruited locally. Case-patients 2 and 3 were confirmed to have JSF on the basis of a ≥4-fold increase in *R. rickettsii*–specific and *R. japonica–*specific IgG titers between acute and convalescent phase serum (Appendix Table 3). At baseline, 12/100 (12%) of the healthy local donors tested positive (range, 1:128–1:1,024; geometric mean, 512) for *R. japonica*–specific IgG.

We measured cytokine and chemokine levels in the serum samples collected from the JSF patients (during acute phase) and 6 healthy donors (Appendix Table 4). The levels of interferon (IFN) γ, interleukin (IL) 6, IL-10, IL-1α, macrophage inflammatory protein 1β, IL-8, IFN gamma-induced protein 10, and monocyte chemoattractant protein 1 in the 4 surviving JSF patients were significantly higher than in the healthy donors (p <0.01), consistent with previous reports, except for the exclusion of tumor necrosis factor α (*12*–*14*). In the case-patient who died, serum levels of IL-6, IL-10, and IFN-γ were 10-fold higher than those in the surviving case-patients and the levels of IL-4, INF-α, granulocyte-macrophage colony-stimulating factor, monocyte chemoattractant protein 1, macrophage inflammatory protein 1β, and IP-10 were 2-fold higher, suggesting that *R. japonica* infection might cause an unregulated hyperinflammatory state, potentially leading to cytokine release syndrome (*14*).

## Conclusion

We identified 5 cases of JSF, including 1 in which the patient died, in Zigui County in the Three Gorges Area of China, where JSF has not previously been identified. Furthermore, our study revealed a high prevalence (12%) of *R. japonica* among residents, suggesting a new endemic area for JSF in China and indicating that JSF might be more widespread than previously thought. We should be alert to the potential risk for JSF, especially in areas where *R. japonica* is detected in vectors (Appendix Figure 1). The JSF cases were confirmed by PCR detection and serologic tests. A strain of *R. japonica* isolated from the blood of the patient who died was revealed to be most closely related to strains LA16/2015 and LA4/2015 detected in Zhejiang Province, suggesting that a virulent strain of *R. japonica* might have spread widely across China.

Delayed treatment is one of the worst prognostic factors for patients with JSF, and as a neglected infectious disease, it might not be considered during differential diagnosis. In our study, the patient who died manifested a faint rash, but without eschar, which resulted in delayed diagnosis and provision of correct antimicrobial treatment when she first visited the rural clinic. Profiling the patient’s serum cytokine and chemokine levels indicated notably elevated IL-6, IL-10, and IFN-γ, characteristic of potential cytokine release syndrome. The primary findings on patient cytokines levels benefit understanding of immune response to *R. japonica* infection. 

Our findings highlight the threat of JSF to public health in China. Healthcare workers, especially in rural areas where residents are at increased risk for tick exposure, should be aware of this potentially deadly infectious disease. Long-term surveillance and investigation of local hosts and vectors of *R. japonica* are necessary to improve the prevention and treatment of JSF.

Appendix Additional information from case-series study of Japanese spotted fever in China.
