# Supplementary material for: Clinical Forms of Japanese Spotted Fever from Case-Series Study, Zigui County, Hubei Province, China, 2021
Source: Emerg Infect Dis. 2023 Jan;29(1):202–6. doi: 10.3201/eid2901.220639 (PMC9796216; doi:10.3201/eid2901.220639)
Supplement: Appendix — Additional information from case-series study of Japanese spotted fever in China. [file 22-0639-Techapp-s1.pdf]

Article DOI: https://doi.org/10.3201/eid2901.220639

Clinical Forms of Japanese Spotted Fever from Case-Series Study, Zigui County, Hubei Province, China, 2021

Appendix

Materials and Methods

Molecular Diagnosis and Amplicon Sequencing

For molecular diagnosis, the DNA extracted from each blood sample was first screened for the presence of zoonotic pathogens (*Rickettsia* spp., *Orientia tsutsugamushi*, *Anaplasma* spp., *Ehrlichia* spp., *Borrelia* spp., *Bartonella henselae*, *Francisella* spp., *Coxiella burnetii*, *Chlamydia psittaci*) by real-time PCR (*1–6*). After other pathogens were ruled out, the DNA samples was subjected to PCR analysis using primer pairs designed to target conserved rickettsial species genes (*glt*A, *omp*A, *omp*B, *sca*4, and 17kD) (Appendix Table 1) (*7–9*). The resulting PCR products were sequenced by Sanger sequencing and sequences were aligned against the GenBank database using the "BLAST" tool (https://blast.ncbi.nlm.nih.gov/Blast.cgi). Phylogenetic trees were generated by the maximum likelihood tree method implemented in MEGA 6 software (http://www.megasoftware.net) with 1000 bootstrap replicates. A one-step RT-nested PCR method was also conducted to further test for severe fever with thrombocytopenia syndrome virus (SFTSV) specific RNA (*10*).

Rickettsial Isolation from the Blood Samples

A 12-well plate was seeded with Vero cells (2.5 × 10^5/well) suspended in minimal essential medium (DMEM) (Gibco, USA) supplemented with 2% fetal bovine serum (Gibco,

USA) and 2 mM glutamine (Sigma, USA) two days before infection. The blood clot homogenate was inoculated into each well (50µL/well), and then the plate was centrifuged in bucket rotors at 700×g for 30 minutes. The plate was incubated at 32°C and 5% CO_2 for 3-5 days. When the culture medium was renewed every 4 days, the cells in each well were scratched manually with a streaking loop. The obtained cells were smeared on a slide and stained by Gimenez staining (Solarbio, China).

Isolation of DNA

Genomic DNA of isolated Rickettsia strain was prepared as previously described (*11*). Mainly, rickettsiae was first released from infected Vero cells by a tissue grinder. Host cell debris was removed by centrifugation at 270 rcf for 5 minutes, and the supernatant was filtered through a 2.0 µm filter unit (Jinteng, Tianjin, China). Rickettsiae were recovered from the filtrate by centrifugation (17,000 rcf, 15 min 4°C) and resuspended in D-PBS with calcium and magnesium (Beyotime, Shanghai, China). The free host DNA were digested by treating with DNase I (15 µg/ml; from bovine pancreas Type II-S, Sigma-Aldrich) for 30 min at room temperature. After DNase I treatment rickettsiae were centrifuged again (17,000 rcf, 15 min 4°C) and genomic DNA was extracted using QIAamp Tissue kit (QIAGEN, USA) and quantified using a Qubit fluorometer (Life Technologies, Paisley, UK). The DNA sample was aliquoted and stocked at –80°C until use. The whole genome of *R. japonica* str. YC21 was sequenced using MinION Nanopore (Oxford Nanopore Technologies, Oxford, UK) and Illumina Hiseq (Illumina, San Diego, CA, USA) methods.

MinION Sequencing

The library was prepared for MinION Nanopore sequencing using a Genomic DNA ligation kit (SQK-LSK109; Oxford Nanopore Technologies) according to the manufacture's protocols. DNA libraries were loaded onto FLO-MIN106 flow cells (R9.4.1), and sequenced using the MinION Mk1C for 72 h.

Illumina Sequencing

The genomic DNA was randomly sheared into short fragments. The obtained fragments were end repaired, A-tailed, and further ligated with Illumina adapter. The short-insert (≈500 bp) libraries were constructed as described in Illumina library preparation kit. The library was checked with Qubit and real-time PCR for quantification and bioanalyzer for size distribution detection. The constructed library was sequenced on an Illumina iSeq 100 platform (San Diego, CA, USA). The raw reads were subsequently trimmed for quality using Trimmomatic (v.0.35) (*12*) with the parameters "ILLUMINACLIP: TruSeq3-PE.fa:2:30:10 LEADING: 3 TRAILING: 3 SLIDINGWINDOW: 4:15 MINLEN: 36". The clean data obtained from this process were used for subsequent analysis.

Genome Assembly

Base calling of the fast5 files was performed using GUPPY (version 1.4.3-1; Oxford Nanopore Technologies). Reads were then BLAST searched against the NCBI nucleotide (nt) database (*13*). All long reads related to the genus *Rickettsia* were mapped to the reference *Rickettsia japonica* str. YH genome sequence (GenBank accession number NC_016050) using Minialign 0.5.3 (*14*) and coverage plots were visualized using Geneious v11.1 (*15*). To improve the accuracy of our assembly, the whole-genome Illumina short reads were mapped to the Oxford Nanopore long reads using BWA-MEM and errors were corrected (*16*). Rawdata was submitted to National Genomics Data Center (https://ngdc.cncb.ac.cn/gsa/browse/CRA006321).

Phylogenetic Analysis of *Rickettsia japonica*

Genome sequences of 34 *Rickettsia japonica* strains were downloaded from the NCBI. Snippy (v4.6.0) (*17*) was used to finds SNPs between the YC21 and the reference genomes to generate a core SNP alignment. The recombinant regions were filtered with Gubbins (v2.4.1) (*18*). Maximum likelihood phylogenetic trees were estimated using FastTree (v.2.1.10) (*19*).

Medication of Patients

Case 1 first visited the village infirmary, complaining of a high fever, where she received an intravenous infusion of cefuroxime (1500 mg bid.), levofloxacin (400 mg qd), and ribavirin (500 mg qd). For the next two days, the dose of ribavirin was increased to 600–700 mg qd. She was then referred to the emergency ward of our hospital and treated with an intravenous infusion of ceftazidime (2000 mg q12h) and gastric administration of minocycline (100 mg qd). The patient died of multiple organ failure 2 days after admission.

Three patients (Cases 2, 4 and 5) received doxycycline (first dose 200 mg po, followed by 100 mg q12h). Case 3 received minocycline (first dose 200 mg po, followed by 100 mg tid).

Determination of Cutoff Values for IFA for JSF Diagnosis

R. japonica str. YC21 was co-cultivated in Vero cell culture to prepare antigen using T75 vented flasks and DMEM supplemented with 2% FBS. *R. japonica* str. YC21 co-cultured in Vero cells was grown in vented tissue culture flasks at 32ºC in an incubator with 5% CO_2. Briefly, when gross cytopathic effect was evident, we harvested infected cells by 0.25% trypsin digestion. The cell concentration was adjusted to $1.2–1.8\times10^5$/mL with DMEM medium containing 2% FBS. Twenty microliters of the cell suspension was added to each well of 12-well slides, and the slides were then air-dried, fixed in acetone and stored at –20°C until they were used.

Sera from 30 anonymous healthy donors from Beijing (no JSF case reported), China and 15 JSF patients (collected by our team and diagnosed via PCR) were used to determine the cutoff values for IFA. The cut-off value was determined following the previous reports (*20*).

References

1. Sprong H, Fonville M, Docters van Leeuwen A, Devillers E, Ibañez-Justicia A, Stroo A, et al. Detection of pathogens in *Dermacentor reticulatus* in northwestern Europe: evaluation of a high-

throughput array. Heliyon. 2019;5:e01270. PubMed https://doi.org/10.1016/j.heliyon.2019.e01270

2. Liu J, Ochieng C, Wiersma S, Ströher U, Towner JS, Whitmer S, et al. Development of a TaqMan array card for acute-febrile-illness outbreak investigation and surveillance of emerging pathogens, including Ebola virus. J Clin Microbiol. 2016;54:49–58. PubMed https://doi.org/10.1128/JCM.02257-15

3. Wolff BJ, Morrison SS, Pesti D, Ganakammal SR, Srinivasamoorthy G, Changayil S, et al. *Chlamydia psittaci* comparative genomics reveals intraspecies variations in the putative outer membrane and type III secretion system genes. Microbiology (Reading). 2015;161:1378–91. PubMed https://doi.org/10.1099/mic.0.000097

4. Versage JL, Severin DD, Chu MC, Petersen JM. Development of a multitarget real-time TaqMan PCR assay for enhanced detection of *Francisella tularensis* in complex specimens. J Clin Microbiol. 2003;41:5492–9. PubMed https://doi.org/10.1128/JCM.41.12.5492-5499.2003

5. Tantibhedhyangkul W, Wongsawat E, Silpasakorn S, Waywa D, Saenyasiri N, Suesuay J, et al. Use of multiplex real-time PCR to diagnose scrub typhus. J Clin Microbiol. 2017;55:1377–87. PubMed https://doi.org/10.1128/JCM.02181-16

6. Calchi AC, Vultão JG, Alves MH, Yogui DR, Desbiez ALJ, De Santi M, et al. *Ehrlichia* spp. and *Anaplasma* spp. in Xenarthra mammals from Brazil, with evidence of novel '*Candidatus anaplasma* spp.' Sci Rep. 2020;10:12615. PubMed https://doi.org/10.1038/s41598-020-69263-w

7. Ishikura M, Ando S, Shinagawa Y, Matsuura K, Hasegawa S, Nakayama T, et al. Phylogenetic analysis of spotted fever group rickettsiae based on *glt*A, 17-kDa, and *rOmp*A genes amplified by nested PCR from ticks in Japan. Microbiol Immunol. 2003;47:823–32. PubMed https://doi.org/10.1111/j.1348-0421.2003.tb03448.x

8. Park HS, Lee JH, Jeong EJ, Kim JE, Hong SJ, Park TK, et al. Rapid and simple identification of *Orientia tsutsugamushi* from other group rickettsiae by duplex PCR assay using *groEL* gene.

Microbiol Immunol. 2005;49:545–9. PubMed https://doi.org/10.1111/j.1348-0421.2005.tb03760.x

9. Teng Z, Shi Y, Peng Y, Zhang H, Luo X, Lun X, et al. Severe case of rickettsiosis identified by metagenomic sequencing, China. Emerg Infect Dis. 2021;27:1530–2. PubMed https://doi.org/10.3201/eid2705.203265

10. Hwang J, Kang JG, Oh SS, Chae JB, Cho YK, Cho YS, et al. Molecular detection of severe fever with thrombocytopenia syndrome virus (SFTSV) in feral cats from Seoul, Korea. Ticks Tick Borne Dis. 2017;8:9–12. PubMed https://doi.org/10.1016/j.ttbdis.2016.08.005

11. Felsheim RF, Kurtti TJ, Munderloh UG. Genome sequence of the endosymbiont *Rickettsia peacockii* and comparison with virulent *Rickettsia rickettsii*: identification of virulence factors. PLoS One. 2009;4:e8361. PubMed https://doi.org/10.1371/journal.pone.0008361

12. Bolger AM, Lohse M, Usadel B. Trimmomatic: a flexible trimmer for Illumina sequence data. Bioinformatics. 2014;30:2114–20. PubMed https://doi.org/10.1093/bioinformatics/btu170

13. Altschul SF, Gish W, Miller W, Myers EW, Lipman DJ. Basic local alignment search tool. J Mol Biol. 1990;215:403–10. PubMed https://doi.org/10.1016/S0022-2836(05)80360-2

14. Suzuki H, Kasahara M. Introducing difference recurrence relations for faster semi-global alignment of long sequences. BMC Bioinformatics. 2018;19(Suppl 1):45. PubMed https://doi.org/10.1186/s12859-018-2014-8

15. Kearse M, Moir R, Wilson A, Stones-Havas S, Cheung M, Sturrock S, et al. Geneious Basic: an integrated and extendable desktop software platform for the organization and analysis of sequence data. Bioinformatics. 2012;28:1647–9. PubMed https://doi.org/10.1093/bioinformatics/bts199

16. Li H, Durbin R. Fast and accurate short read alignment with Burrows-Wheeler transform. Bioinformatics. 2009;25:1754–60. PubMed https://doi.org/10.1093/bioinformatics/btp324

17. Seemann T. Snippy. Fast bacterial variant calling from NGS reads. 2015 [cited 2021 Dec 8] https://github.com/tseemann/snippy

18. Croucher NJ, Page AJ, Connor TR, Delaney AJ, Keane JA, Bentley SD, et al. Rapid phylogenetic analysis of large samples of recombinant bacterial whole genome sequences using Gubbins. Nucleic Acids Res. 2015;43:e15. PubMed https://doi.org/10.1093/nar/gku1196

19. Nguyen LT, Schmidt HA, von Haeseler A, Minh BQ. IQ-TREE: a fast and effective stochastic algorithm for estimating maximum-likelihood phylogenies. Mol Biol Evol. 2015;32:268–74. PubMed https://doi.org/10.1093/molbev/msu300

20. Villumsen S, Jørgensen CS, Smith B, Uldum S, Schiellerup P, Krogfelt KA. Determination of new cutoff values for indirect immunofluorescence antibody test for Q fever diagnosis in Denmark. Diagn Microbiol Infect Dis. 2009;65:93–8. PubMed https://doi.org/10.1016/j.diagmicrobio.2009.06.004

Appendix Table 1. Primers for PCR and sequencing

Primer	Target gene	Sequence (5'-3')	References‡
RpCS780p*	*glt*A	GACCATGAGCAGAATGCTTCT	*(7)*
RpCS1258n*		ATTGCAAAAAGTACAGTGAAC	
RpCS877p†		GGGGGCCTGCTCACGGCGG	
RpCS1258n†		ATTGCAAAAAGTACAGTGAAC	
Rr190k.71p*	*omp*A	TGGCGAATATTTCTCCAAAA	*(7)*
Rr190k.720n*		TGCATTTGTATTACCTATTGT	
Rr190k.71p†		TGGCGAATATTTCTCCAAAA	
Rr190k.602n†		AGTGCAGCATTCGCTCCCCCT	
Rgroel-OF*	*groEL*	AAGAAGGMGTGATAAC	*(8)*
Rgroel-OR*		ACTTCMGTAGCACC	
Rgroel-IF†		GATAGAAGAAAAGCAATGATG	
Rgroel-IR†		CAGCTATTTGAGATTTAATTTG	
R005F	*omp*B	GTAACCGGAAGTAATCGTTTCGTAAA	*(9)*
R005R		CTTTATAACCAGCTAAACCACCTT	
R010F	17kDa	GCTCTTGCAACTTCTATGTTACA	*(9)*
R010R		CATTGTTCGTCAGGTTGGCGgCATG	
R011F	*sca*4	ATGAGTAAAGACGGTAACCT	*(9)*
R011R		AAGCTATTGCGTCATCTCCG	

*Nested PCR primers, 1st round

†Nested PCR primers, 2nd round

‡Appendix Reference

Appendix Table 2. Virulence-associated genes predicted from *Ricettsia japonica* YC21 genome

VFclass	Virulence factors	Related genes
Actin-based motility	RickA	*rick*A
	Sca2	*sca*2
Adherence and invasion	Adr1	*adr*1
	Adr2	*adr*2
	Sca1	*sca*1
	Sca4	*sca*4
	rOmpA/Sca0	*omp*A
	rOmpB/Sca5	*omp*B
Enzyme	Hemolysin C	*tly*C
	Phospholipase A2	*pat*1
		*pat*2
	Phospholipase D	*pld*
Secretion system	Rvh T4SS effector	*ral*F
	Rvh T4SS	*rvh*B10
		*rvh*B11
		*rvh*B1
		*rvh*B2
		*rvh*B3
		*rvh*B4a
		*rvh*B4b
		*rvh*B6a
		*rvh*B6b
		*rvh*B6c
		*rvh*B6d
		*rvh*B6e
		*rvh*B8a
		*rvh*B8b
		*rvh*B9a
		*rvh*B9b
		*rvh*D4

Appendix Table 3. Serum immunoglobulin antibody titers of the Japanese spotted fever cases*

	Time of sample collection†		IgM (AP)			IgG (AP/CP)		
Cases	AP	CP	*R. rickettsii*	*R. typhi*	*R. japonica*‡	*R. rickettsii*	*R. typhi*	*R. japonica*‡
Case 1	10	NA	ND	ND	ND	ND/NA	ND/NA	ND/NA
Case 2	7	30	64	ND	64	64/512	ND/128	128/1024
Case 3	10	30	256	64	256	1024/4096	512/1024	1024/>4096
Case 4	14	NA	128	ND	256	1024/NA	128/NA	2048/NA
Case 5	10	NA	128	ND	256	2048/NA	512/NA	2048/NA

*AP, acute phase; CP, convalescent phase; IFA, immunofluorescence assay; NA, not available; ND, none detected

†Days after first symptom onset

‡Coating with isolated *R. japonica* str. YC21

Appendix Table 4. Serum cytokine and chemokine concentrations (ng/µL) *

		Case-patients†		Control participants			
Type	Identity	Mean	SD	Mean	SD	p-value‡	Case-patient 1
Cytokines	IFN-γ	28.95	0.40	20.20	5.10	<0.01	417.67
	TNF-α	47.55	7.28	69.31	23.50		85.86
	IL-8(CXCL-8)	674.35	291.80	19.27	12.42	<0.01	585.05
	IL-12p70	12.45	2.01	18.49	3.61		23.80
	IL-1β	1.92	0.15	2.07	0.49		3.93
	IL-4	13.51	2.77	10.66	2.64		34.02
	IL-6	31.93	5.46	5.10	1.24	<0.01	647.17
	IL-17A(CTLA-8)	11.20	2.55	17.68	4.81		16.23
	IL-10	22.70	2.24	0.89	0.37	<0.01	406.85
	IL-13	3.87	1.10	6.00	1.50		3.52
	GM-CSF	5.16	0.32	6.49	0.29	<0.01	10.61
	IFN-α	2.27	0.45	2.41	0.70		5.81
	IL-1α	12.02	4.83	2.81	3.64	<0.01	13.55
Chemokines	MIP-1α(CCL3)	101.46	70.32	31.12	20.11		104.25
	MIP-1β	504.99	281.83	110.70	36.49	<0.01	935.94
	IP-10(CXCL10)	431.79	28.04	27.56	15.62	<0.01	961.01
	MCP-1(CCL2)	53.29	30.36	112.60	16.27	<0.01	226.69

*SD, standard deviation

†Four surviving case-patients (2, 3, 4, and 5).

‡Four surviving case-patients (2, 3, 4, and 5) compared with controls analyzed using a paired Student’s t-test in Microsoft Excel.

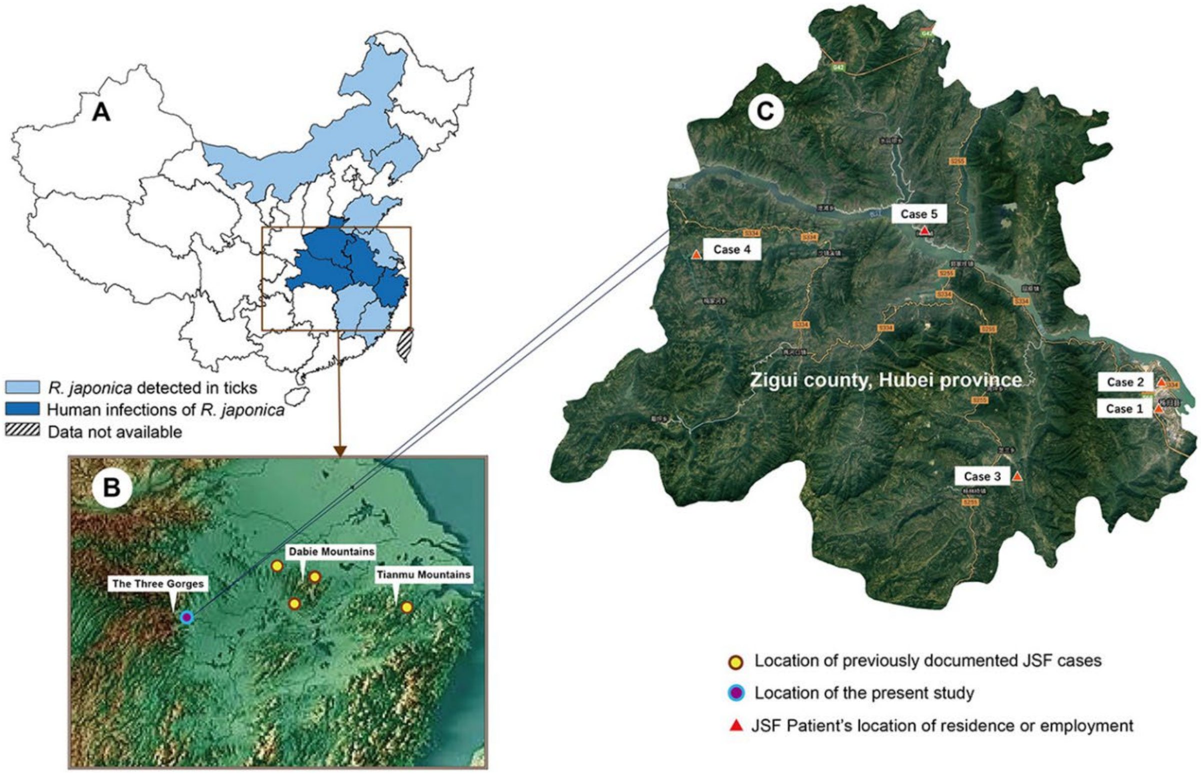

Appendix Figure 1. *Rickettsia japonica* in mainland China. A) Distribution of *Rickettsia japonica.* Provinces where *R. japonica* has not been detected are white, if detected in ticks, light blue, if from human-related samples, deep blue. Out of 34 provinces and special administrative regions, 10 (29.4%) are *R. japonica*–positive, and 4 provinces had human infections. B) Japanese spotted fever cases found in China. The reported Japanese spotted fever cases in the present work were in the Three Gorges Area, a mountainous area with large water systems. Previously documented Japanese spotted fever cases in China were distributed in the Dabie and Tianmu Mountains. C) Map of Zigui County. Most cases were from the river valley area.

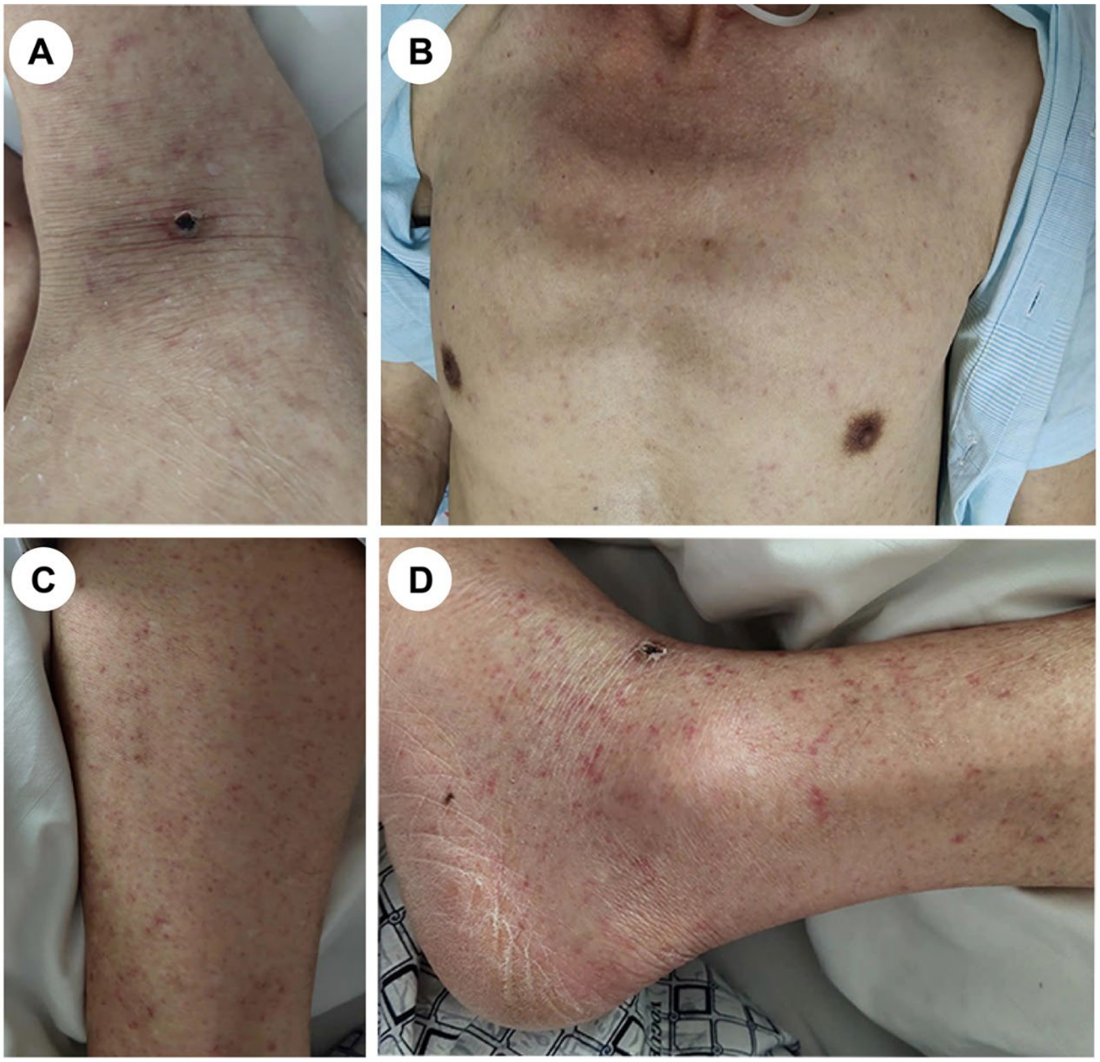

Appendix Figure 2. A) Eschar on the left upper ankle; pink-red spotted rashes on B) the chest, C) calves, and D) left foot of a patient with Japanese spotted fever (case-patient 3)

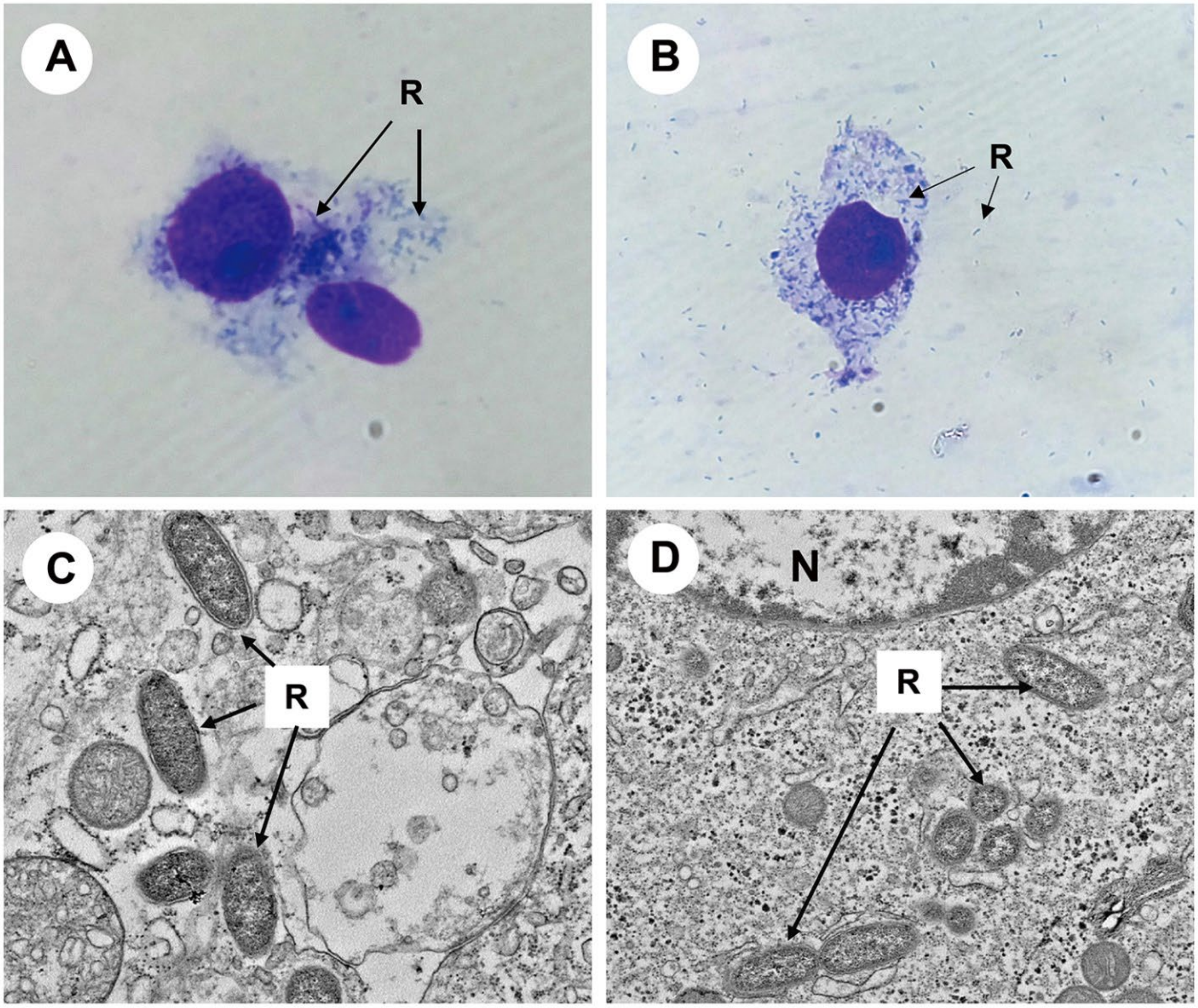

Appendix Figure 3. Photomicrographs of cells infected with *Rickettsia japonica* strain YC21 isolated from blood of case-patient 1. A) and B) giemsa-stained Vero cells under a light microscope; C) and D) Vero cells infected with *R. japonica* strain YC21 under a scanning electron microscope.

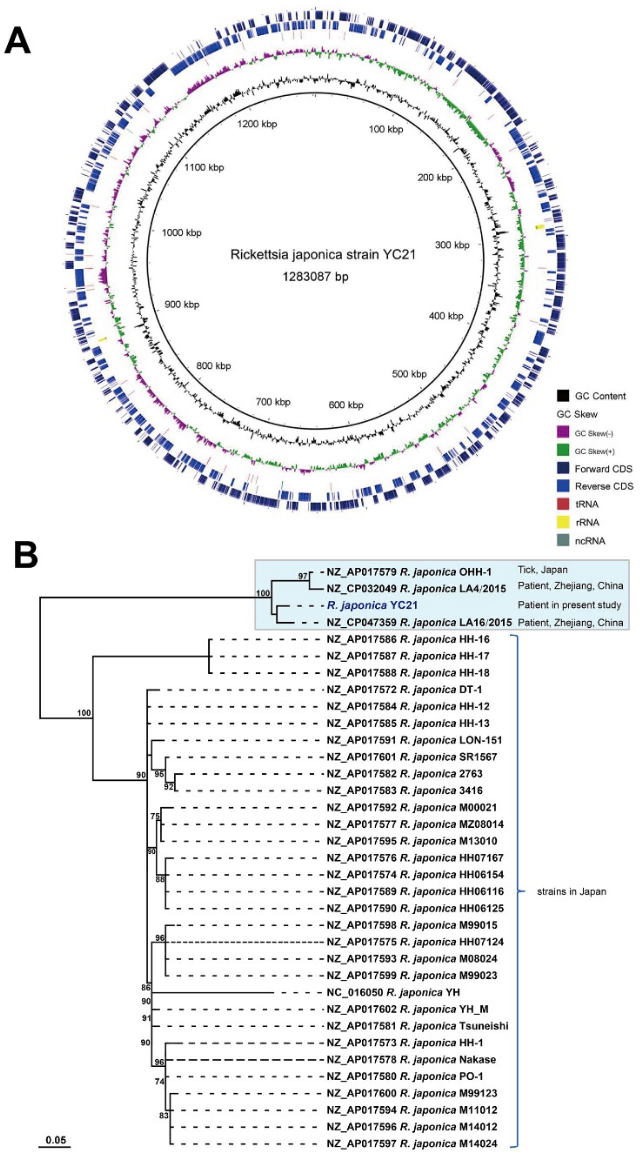

Appendix Figure 4. Genome of *Rickettsia japonica* strain YC21 is most closely related to that of *Rickettsia japonica* strain LA16/2015. A) Genome of *R. japonica* strain YC21. Whole genomic sequence of *R. japonica* strain YC21 was 1,283,531 bases with 32.4% GC content. Structural annotation of the genome revealed a total of 1,439 coding sequences and 40 RNAs consisting of 33 transfer RNAs, 3 ribosomal RNAs (23S, 16S and 5S rRNA), 3 noncoding RNAs, and 1 transfer-messenger RNA; B) Phylogenetic tree for core genes of *R. japonica* strains. An unrooted phylogenetic tree generated on the basis of the genome shows the evolutionary relationships across *R. japonica* strains. Scale bar represents nucleotide distance.
